# Ecological study on seaweed diversity in Suez, Hurghada and Marsa Alam, Red Sea, Egypt

**DOI:** 10.1186/s12862-025-02389-5

**Published:** 2025-05-26

**Authors:** Mahmoud Sami, Fayrouz Ahmed, Tarek A. Temraz, Amira A. Ali

**Affiliations:** Department of Marine Science, Faculty of Science, Suez Canal University, Ismailia, Egypt

**Keywords:** Seaweeds vegetation, Functional groups, Ecological study, Marine ecosystems, Diversity, Red sea

## Abstract

Seaweed vegetation is widely distributed along the Red Sea coasts. Therefore, the current study presents an ecological study on the spatial and temporal variations of seaweed vegetation at three different sites (Suez, Hurghada and Marsa Alam) along the western coast of the northern Red Sea. The study was conducted through regular seasonal visits over four seasons, starting from winter 2022. Physicochemical parameters were measured, and the coverage of seaweed species was estimated using the quadrat method. Forty-seven species of seaweeds were collected and identified from the studied sites during the study period. Site II (Hurghada) had the highest number of species (*n* = 37), whereas site I (Suez) had the lowest (*n* = 11). The findings reveal significant variations in species composition, and coverage, highlighting the influence of environmental factors and seasonal changes on seaweed communities**.** Site I (Suez) recorded the highest average percentage cover of Chlorophyta (97%), where Phaeophyceae (50%) and Rhodophyta (38%) recorded the maximum at site II and site III (Marsa Alam), respectively. In general, winter and spring recorded the highest number of species (43 and 38, respectively), while autumn recorded the lowest (*n *= 33). In spring, Chlorophyta recorded the highest coverage (35.7%) mostly represented by *Ulva lactuca, Caulerpa racemosa*, *Dictyosphaeria cavernosa, Valonia aegagropila* and *Cladophora prolifera*, followed by Rhodophyta (34.3%) with a dominance of *Actinotrichia fragilis* and *Jania rubens.* Furthermore, regular biodiversity monitoring is necessary to continuously update the species and detect any changes that may occur in the physicochemical and biological parameters of the ecosystem, including the effects of climate change.

## Introduction

The serious concerns about the destruction of seaweeds resources and changes in the variety of various life forms make it necessary to study the taxonomy and species diversity for better management of marine algae [53]. The floristic composition of aquatic algal flora, distribution, diversity, and periodicity sequence can be used to evaluate ecological changes. This is especially significant as the marine ecosystem has been subjected to considerable alternation in recent decades [32]. Hence, understanding the diversity and distribution of seaweeds in any particular area is important for the sustainable utility of the resource. Additionally, this will aid in the regulation and control of invasive harmful species, the implementation of protective measures for conserving extinct and endangered species and the undertaking of environmental impact assessments [49].

Environmental conditions of variable water currents, and high concentrations of salts and sunlight, have a substantial impact on the growth and chemical composition of the macroalgal community [10]. According to Jung et al. (2013), the physicochemical characteristics of water are primarily regulated by location and season, which in turn determine the occurrence of specific algae at a specific place and time. As a result, studying the physicochemical aspects of the marine ecosystem is extremely crucial. Moreover, it is well known that the habitat may determine the type of algal flora present and allow some species to predominate at the expense of others [33].

Seaweeds in coastal waters are subjected to increased anthropogenic loading as a result of human activities in densely populated and industrialized zones including agriculture, urbanization, and tourism [54]. Such disturbance in the chemical environment leads to alteration in community structure and abundance within the ecosystem [39]. Temperature, hydrogen ion concentration, salinity, and alkalinity are all strong key factors in aquatic ecosystem. The variation in these factors among sites is responsible for the presence of new functional groups of seaweeds, while seaweeds periodicity and standing crop are significantly impacted by seasonal fluctuation [33] and [18].

Research on macroalgae in the Red Sea began in the eighteenth century with Forsskål [28], who gathered several species from the northern region. Since then, marine phycological studies in the Red Sea have advanced significantly. Key contributions to the understanding of Red Sea algae were made by Naser (1947), Zanardini [57], Rayss [47], and Rayss and Dor [48]. By 1968, Papenfuss [42] had reviewed 45 scientific papers while documenting benthic algae from various parts of the Red Sea. Subsequent research has focused on the taxonomic diversity of macroalgae in the northern Red Sea, as seen in works by Aleem [6], El-Manawy and Shafik [24], El-Manawy [22] and Issa et al. [33]. Most recently, Einav et al. [20] provided an updated checklist of Red Sea macroalgae from 1756 to 2020, reporting 576 taxa, including 286 Rhodophyta, 157 Ochrophyta (class Phaeophyceae), and 133 Chlorophyta.

Seaweeds predominate extensive areas in the Red Sea [8, 16, 22, 32]. Following the opening of the Suez Canal, several expeditions to the Indian Ocean sailed over the Red Sea. A substantial number of seaweeds were collected during that time period and identified as Red Sea type species [43]. Consequently, considering the significance of seaweed communities, as both a biodiversity supporter and a rich economic source, it is important to follow up on the variation and distribution in these communities over time and place under the influence of various ecological factors along the Egyptian coasts. The current study presents an ecological study on the spatial and temporal variations of seaweeds vegetation at selected sites along the Western coast of the Egyptian Red Sea.

## Materials and methods

**Fig. 1:**
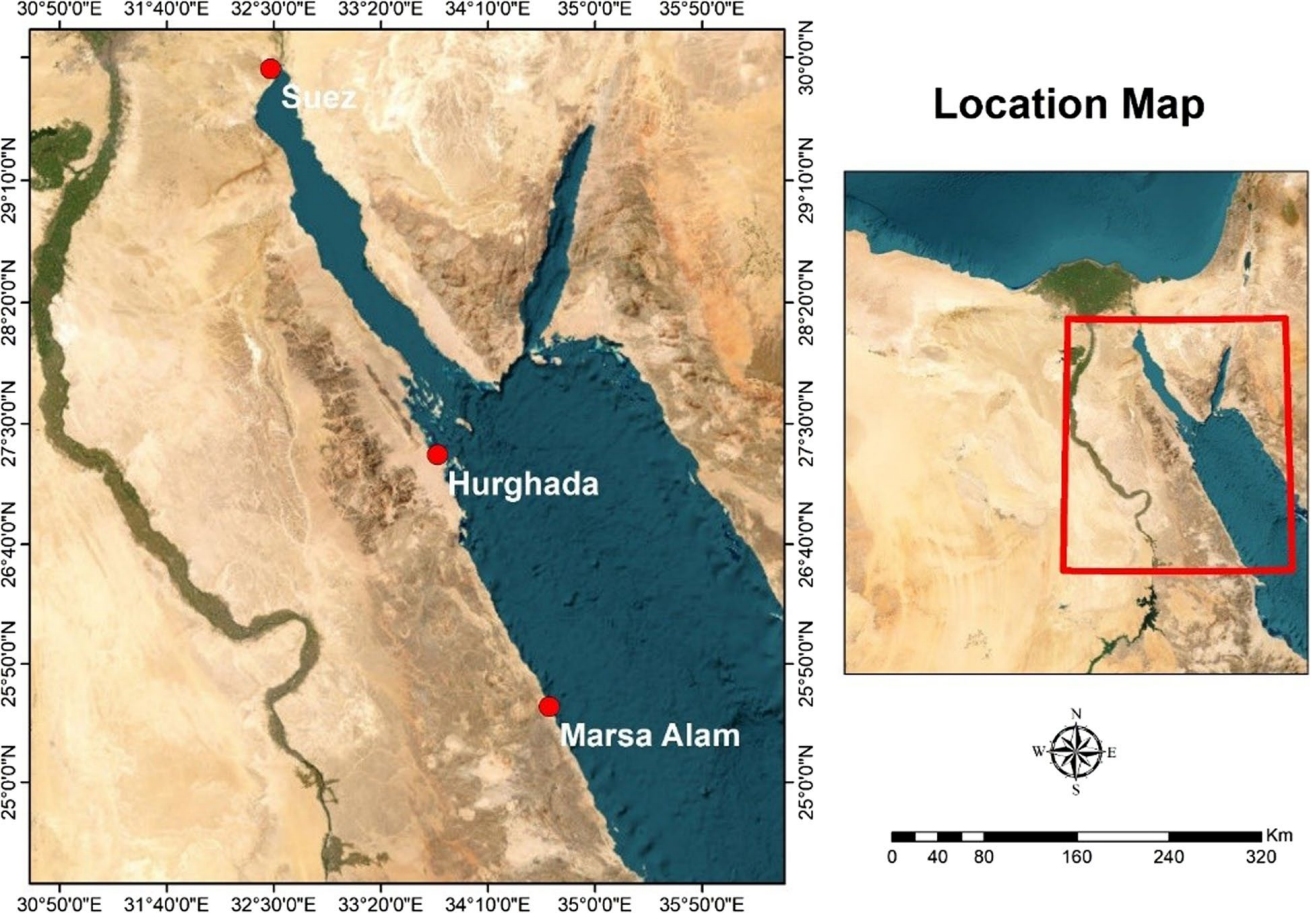
Location of the selected sites along the western coast of the Egyptian Red Sea

Three different sites along the western coast of the northern Red Sea were chosen (Fig. 1). These sites are located in three cities: Suez (29° 55′ 29′′ N, 32° 28′ 31′′ E), Hurghada (27° 17′ 07′′ N, 33° 46′ 17′′ E), and Marsa Alam (25° 32′ 41′′ N, 34° 38′ 30′′ E) and represent different hydrographic habitats. Their proximity to a research laboratory (National Institute of Oceanography and Fisheries (NIOF) in Suez and Hurghada, and the research center of Hurghada Environmental Protection and Conservation Association (HEPCA) in Marsa Alam) was taken into consideration. The accessibility of these laborites and the availability of facilities would aid in the fieldwork.

### The studied sites description

**Fig. 2:**
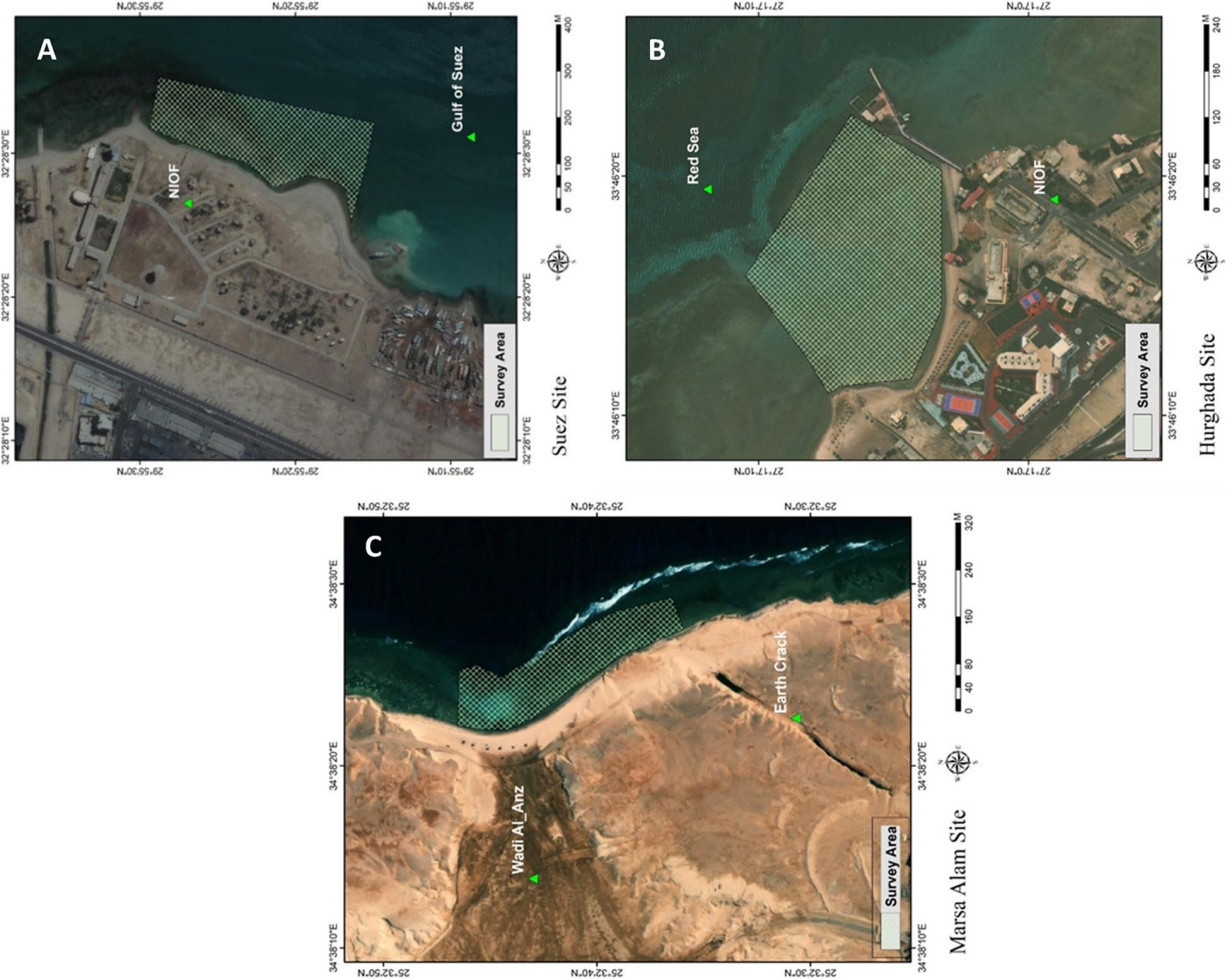
Surveyed areas at different sites. **A** Site I (Suez), **B** Site II (Hurghada) and **C** Site III (Marsa Alam)

Site (I) is located in Suez city in front of the NIOF. This site is characterized by a sandy substrate and a short reef flat. It suffers from pollution mainly due to the discharge of Attaka power station effluents and industrial wastes, in addition to other pollutants produced by the different maritime activities. The current direction is generally anti-clockwise, from north to south (Fig. 2A).

Site (II): It is located in the northern part of Hurghada city, opposite to the NIOF. This site is characterized by a wide and shallow reef flat with many depressions and lagoons (Fig. 2B). Water currents follow the prevailing direction in the Red Sea from north to south. The coastline facing this site is not heavily subjected to the direct effects of anthropogenic activities, but it suffers from underground wastewater seepage and extensively high sedimentation rates throughout the year [2].

Site (III): The site is situated around 60 km north of Marsa Alam in front of the HEPCA research center (Fig. 2C). The width of the reef flat at this site is in the range of 80 to 120 m. The reef is dominated by hard dead and live corals, and much more exposed to wave action. The site is also distinguished by the presence of an earthquake crack of about 300 m in length.

### Duration of the study

The present study was carried out on a basis of regular seasonal visits to the three sites over four seasons starting from winter 2022.

### Field data collection

#### Physicochemical parameters

Air and water temperature (°C), pH, and salinity (‰) were determined seasonally in the field by utilizing the multiparameter instrument (YSI Pro plus). The readings were taken just below the water surface at a depth of 50 cm. For each site, the measurements were repeated three times and the mean value calculated.

Seawater samples were seasonally collected from the selected sites from a depth of 50 cm below the surface and placed in previously acid-washed dark bottles. They were kept deep frozen until the chemical analysis was conducted. Four nutrients including inorganic nitrite (NO_2_), nitrate (NO_3_), inorganic phosphate (PO_4_), and ammonium (NH_4_^+^) were determined using spectrophotometry (Perkin Elmer 2380 model). The measurements were based on the methods described by APHA [12] and the results were expressed as mg/l.

#### Seaweeds vegetation assessment

In order to determine the spatial changes in seaweed assemblages, three line-transects were set in each site. The transects were placed at intervals of 50 m and were perpendicular to the coastline, spanning from the highest point reached by the tides to the outer edge of the reef flat. They varied in length according to the width of the reef flat at each site. This technique is widely used for description and mapping of coral reefs and macroalgae [38]. The coverage and abundance of seaweeds species were estimated using the quadrat method as described by Russell and Fielding [52]. At each transect, at least 10 quadrats were randomly laid, as replicates. The quadrat dimensions were (50 cm × 50 cm) divided into 10 cm subunits separated by strings. The quadrat was placed and flipped on both sides along the transect to cover all species existed. Seaweed vegetation was quantitatively characterized through the visual assessment of the percentage coverage of each species within the quadrat, and then the average was calculated. All works were carried out by snorkeling during high tide based on the tide table of each site. In addition, seaweeds were photographed underwater using a waterproof Nikon COOLPIX P7000 or GoPro HERO6 cameras, whichever available at the time of survey.

#### Seaweeds sampling

Seaweed samples were collected manually. Samples were then washed extensively in seawater to eliminate any epiphytes, sand, or other debris. Thoroughly in seawater to eliminate epiphytes, sand, and other detrital materials. Portion of the samples was preserved in solution of 4% formaldehyde in seawater, while the other was used to make herbarium sheets for preservation and identification purposes. Other cleaned samples were dried in shade and kept in labeled sealed plastic bags including records of site, habitat type and the general ecological conditions for further investigation.

#### Identification of seaweed species

In the laboratory, the collected seaweeds were examined by light microscope and identified based on morphological characteristics such as pigmentation, thallus structure, as well as reproductive and other characters, utilizing identification manuals. The standard references were Nasr [41], Børgesen [15], Dawson [19], lipkin [37], Aleem [5], and Abbott [1]. However, the currently valid and accepted names of the seaweed species were according to the taxonomic database AlgaeBase [30]. Seaweeds were classified into functional groups according to Steneck and Dethier [55] and Rubal et al. [51].

#### Statistical data analysis

Boxplot graphs were used to illustrate the spatial and temporal variability. The size of the boxplot in relation to a specific site represents the variability across different seasons, whereas the size in relation to a particular season indicates the variability across various sites during that season. Two-way ANOVA were used to analyze the variation in the coverage of dominant seaweed species within sites and seasons as well as to identify significant differences between the physicochemical parameters in different sites and seasons. If significant differences were present, post hoc test was employed to check for differences between means. Additionally, Pearson Correlation analysis was used to describe the relationship between the physicochemical parameters and the dominant macroalgal cover. All statistical tests were performed using SPSS (Version 22) software at a significance level of *P* ≥ 0.05.

Multivariate analysis of principle component analysis (PCA) was performed to investigate the relationship between the physicochemical parameters within sites. Cluster analysis used to reveal similarities in seaweed coverage between different sites and seasons. The clusters are represented as dendrogram, where the samples were plotted on vertical axis and the similarity level on horizontal axis. Both analyses were performed using ORIGINPRO software (vesion 2024b). Diversity indices (Shannon–Weaver diversity index, species evenness and species richness) were estimated using PRIMER software (Version 7).

## Results

### Physicochemical parameters of the studied sites

**Fig. 3:**
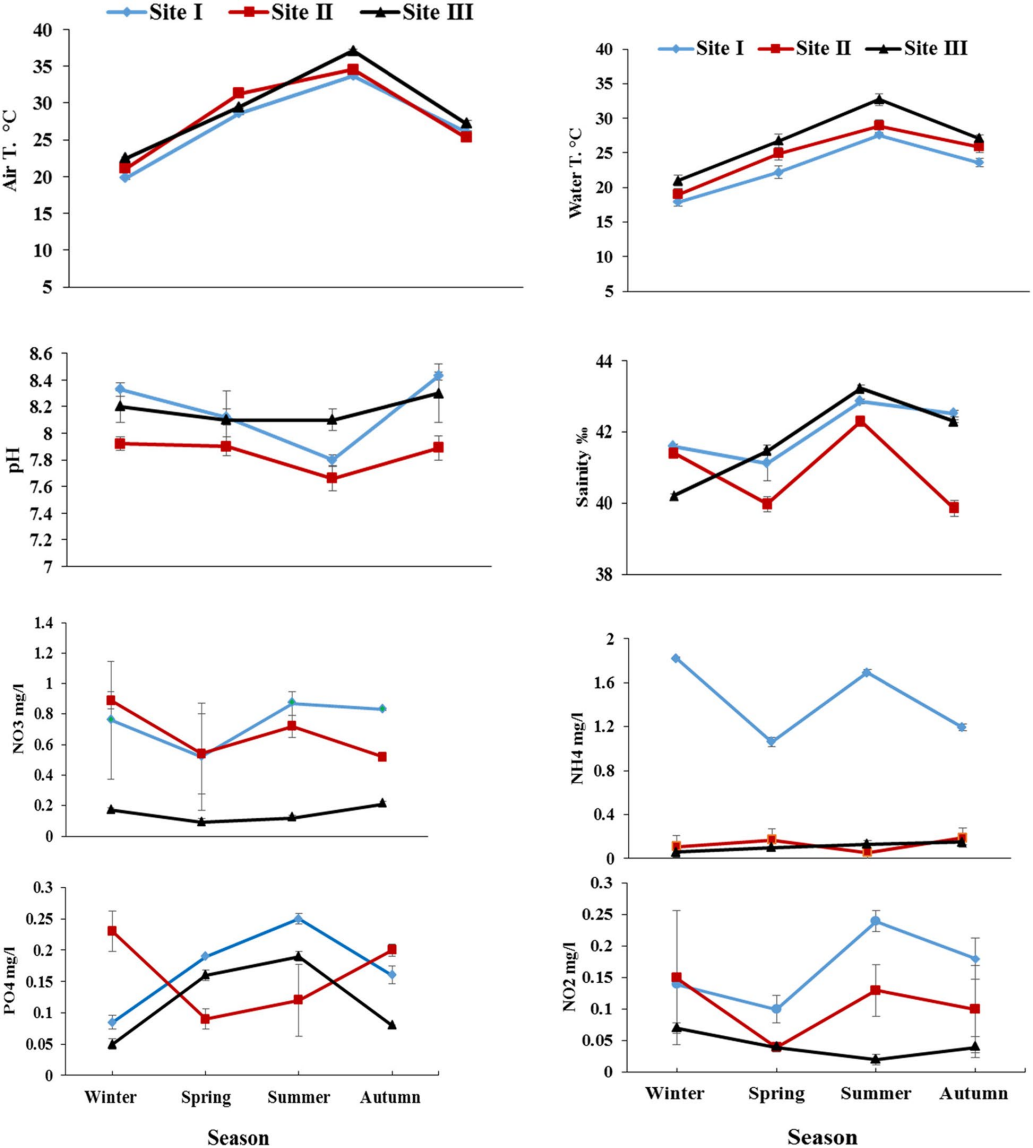
Physicochemical parameters at the studied sites during the four seasons (mean±SD)

Maximum air and water temperatures were 37.1 °C and 32.7 °C, respectively during summer at site III, while the minimum values were recorded at site I (19.8 °C and 17.9 °C, respectively) during winter (Fig. 3). The highest salinity was recorded at site III (43.22‰) during summer, whereas site II recorded the lowest (39.85‰) during autumn. The values of pH varied between 7.66 at site II during summer and 8.43 at site I during autumn.

The highest ammonium concentration was 1.82 mg/l at site I during winter, while site III recorded the lowest (0.06 mg/l) during the same season (Fig. 3). The maximum concentration of nitrate was 0.89 mg/l at site II during winter, while the minimum was 0.09 mg/l at site III during spring. Summer recorded the highest and lowest concentration of nitrite 0.24 and 0.02 mg/l at sites I and III, respectively. The levels of dissolved phosphate differed from one site to another with the maximum (0.25 mg/l) at site I during summer and the minimum (0.05 mg/l) at site III during winter. The physicochemical parameters of seawater varied significantly (*p* < 0.05) between seasons and sites except for nitrate between seasons (*p* = 0.152) (Table 1).

**Table 1:** ANOVA test results for the physicochemical parameters of sites and seasons

Parameter	Air temperature	Water temperature	pH	Salinity	Nitrate (NO_3_)	Ammonium (NH_4_)	Phosphate (PO_4_)	Nitrite (NO_2_)
	***p* value**							
**Site**	0.000^**^	0.000^**^	0.000^**^	0.000^**^	0.000^**^	0.000^**^	0.000^**^	0.000^**^
**Season**	0.000^**^	0.000^**^	0.000^*^	0.000	0.152	0.000^**^	0.001^**^	0.028^*^
Site* Season	0.000^**^	0.066	0.000^**^	0.000^**^	0.468	0.000^**^	0.000^**^	0.098

Where, * = *p* < 0.05; ** = *p* < 0.01

**Fig. 4:**
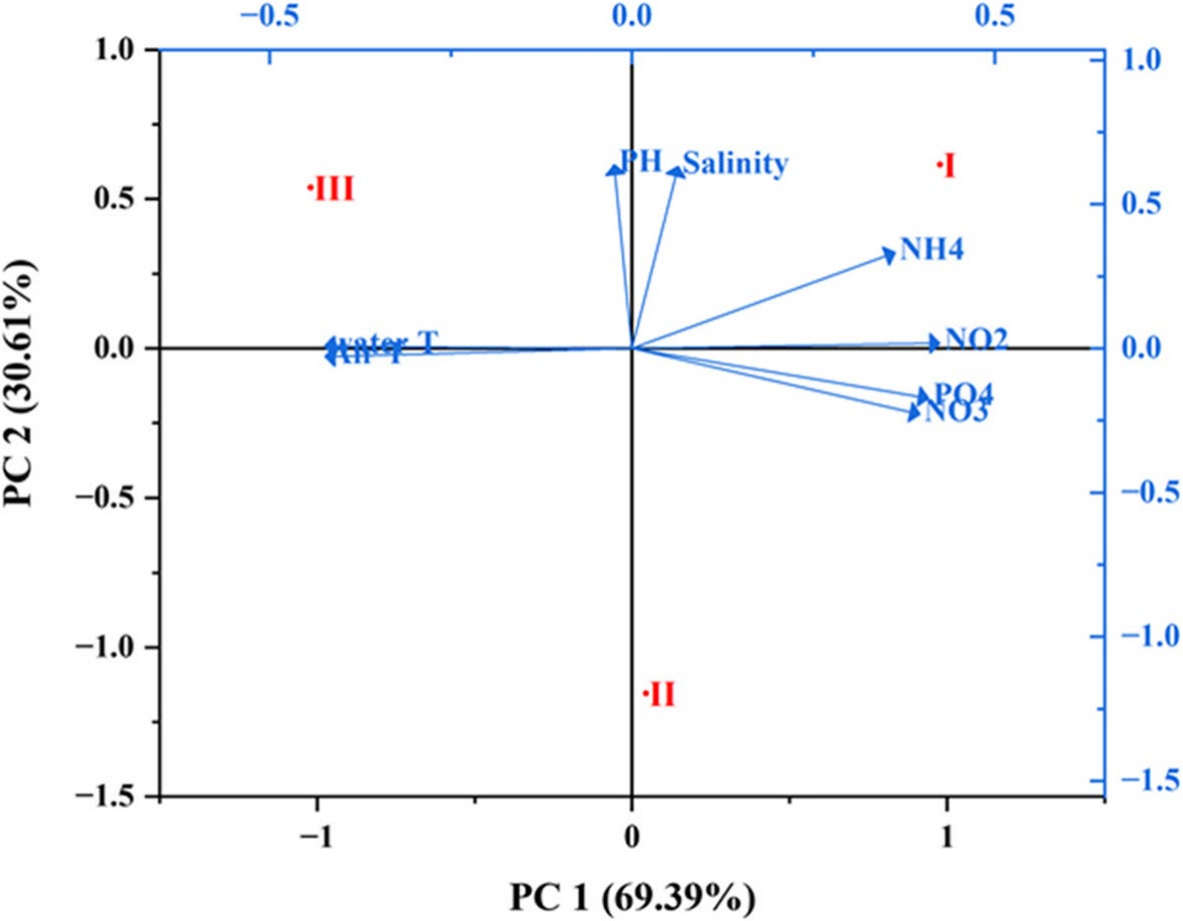
Principle component analysis (PCA) for physicochemical parameters (arrows) with sites (points) during the study period

The output data of the principle component analysis revealed that two main factors (PC1- PC2) affected parameters distributions (Fig. 4). PC1 explained 69.39% of the total data variance and represented positive loading for NH_4_, NO_2_, NO_3_ and PO_4_ (0.36, 0.42, 0.39 and 0.41, respectively). PC2 interprets 30.61% of the total variance including positive loadings for pH (0.64) and salinity (0.63). Dissolved phosphate had the strongest positive correlation with both nitrite and nitrate, while water and air temperatures were negatively correlated with nitrite.

### Seaweeds vegetation

#### Floristic composition

**Fig. 5:**
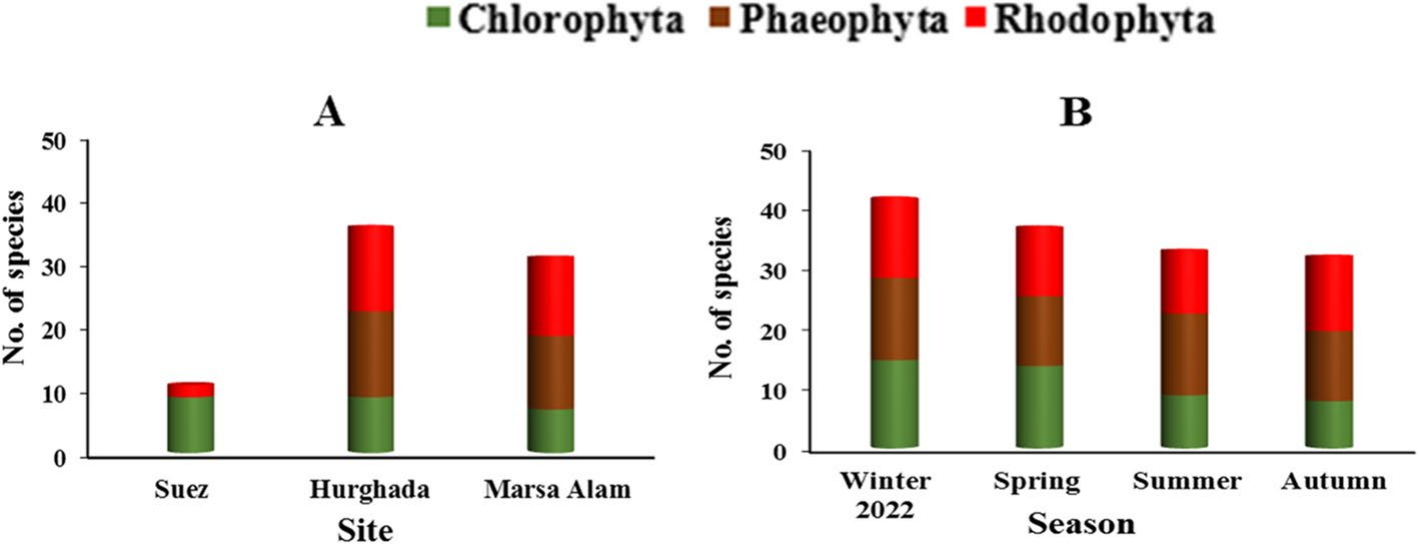
Total number of seaweeds species of the different divisions during the study period in different sites (**A**) and seasons (**B**)

Forty-seven species of seaweeds were identified from the studied sites during the study period. 38% of them (18 species) belong to Chlorophyta, 32% (15 species) to Phaeophyceae and 30% (14 species) to Rhodophyta. Table 2 shows the annual average cover of each species at the three sites. The maximum number of species (*n* = 37) was recorded at site II (Hurghada), while the minimum (*n* = 11) was observed at site I (Suez) (Fig. 5A). In general, winter and spring recorded the highest number of species (43 and 38, respectively). However, autumn recorded the lowest (*n* = 33) (Fig. 5B).

**Table 2:**
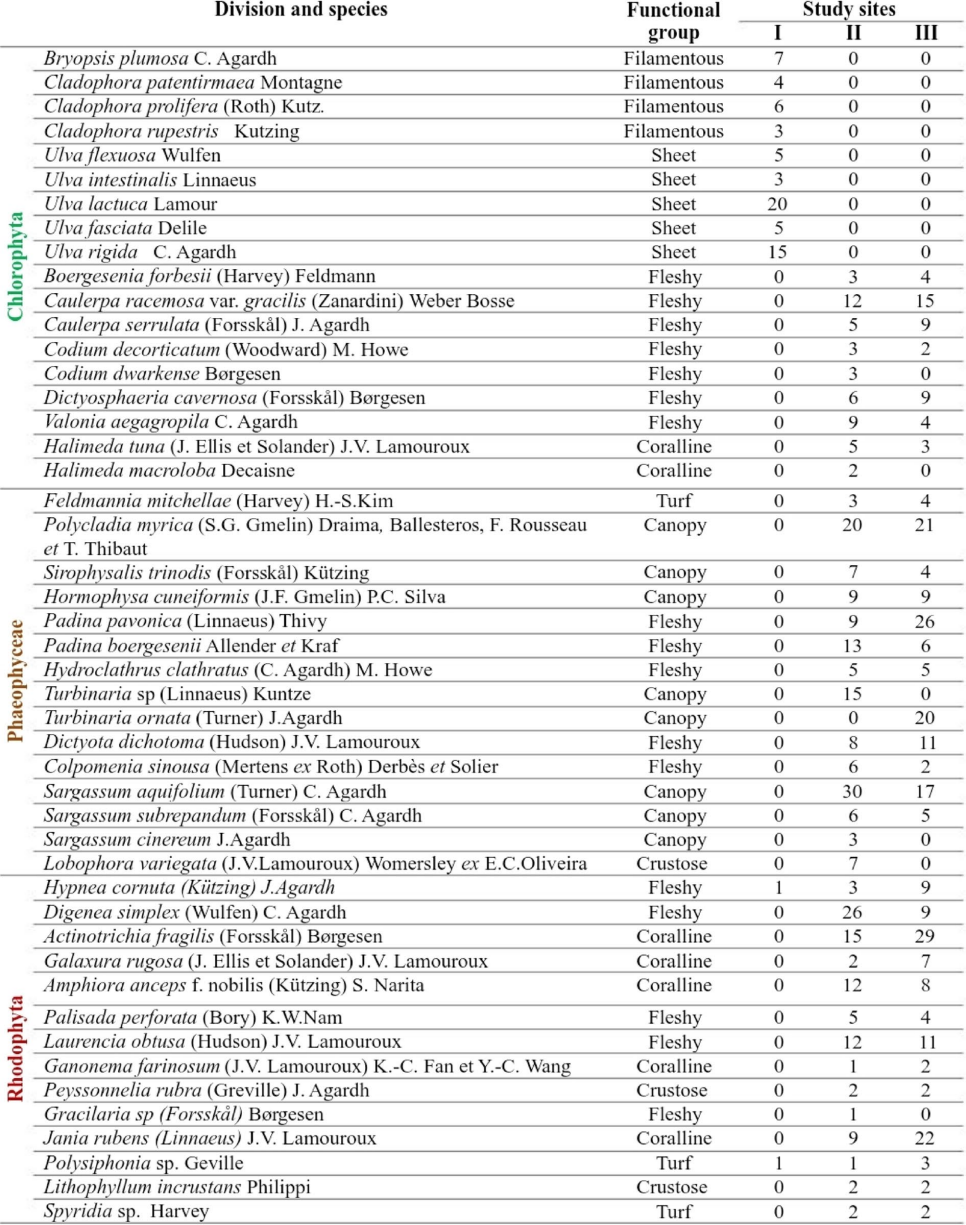
Species list, functional groups, and annual average cover (%) at the studied sites

#### Functional groups diversity

Seven functional groups (small filamentous, turf-forming, sheet, calcified crustose, upright fleshy, coralline forms and canopy) were observed at the surveyed sites during the study period (Table 3). In terms of diversity, Chlorophyta had the highest number of representative groups, followed by Phaeophyceae and then Rhodophyta. Upright fleshy was the most diverse group with 17 species (7 Chlorophyta, 5 Phaeophyceae, and 5 Rhodophyta), followed by 8 species of canopy category, all from Phaeophyceae. Filamentous and sheet groups were only represented in Chlorophyta.

**Table 3:** Functional groups of seaweeds in the three sites during the study period

**Division**	**Functional groups**							**Total**
	**Filamentous**	**Sheet**	**Upright Fleshy**	**Canopy**	**Coralline**	**Crustose**	**Turf**	
**Chlorophyta**	4	5	7	0	2	0	0	18
**Phaeophyceae**	0	0	5	8	0	1	1	15
**Rhodophyta**	0	0	5	0	5	2	2	14
**Total**	4	5	17	8	7	3	3	**47**

#### Diversity indices of seaweeds species at the studied sites

**Fig. 6:**
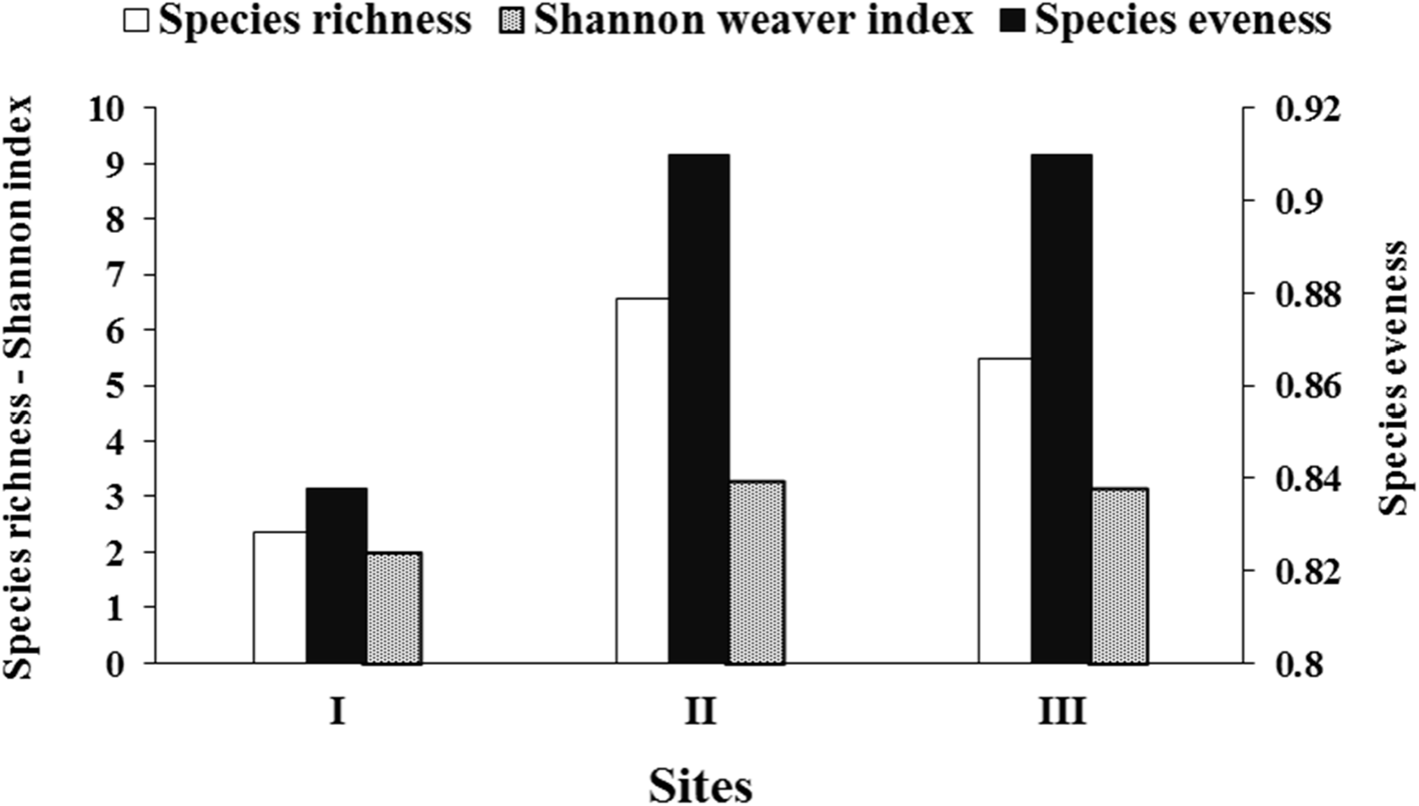
Diversity indices of algal communities in the three sites

Site II recorded the highest value of species richness and Shannon–Weaver index (6.54 and 3.31, respectively), followed by site III (5.49 and 3.17, respectively) (Fig. 6). Species evenness ranged from 0.84 in site I to 0.91 in both sites II and III.

#### Distribution of seaweeds within sites and seasons

**Fig. 7:**
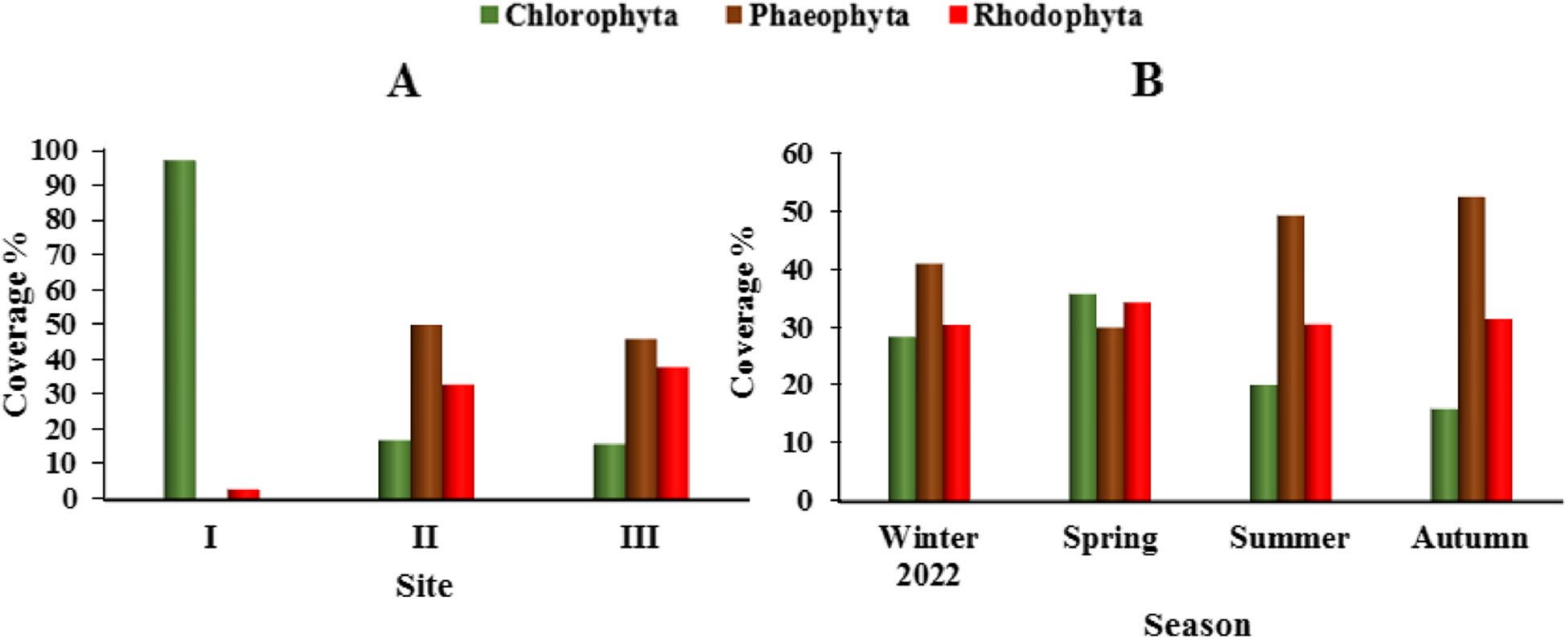
Coverage % (mean±SD) of seaweed divisions during the study period at the different sites (**A**) and seasons (**B**)

Generally, site I recorded the highest average percentage cover of Chlorophyta, where Phaeophyceae and Rhodophyta recorded the maximum at site II and III, respectively (Fig. 7A). Phaeophyceae recorded the maximum coverage during autumn and summer (52.5% and 49.3%, respectively) with a domination of *Polycladia myrica*, *Padina pavonica*, *Dictyota dichotoma*, *Sargassum aquifolium* and *Hormophysa cuneiformis* (Fig. 7B and Table 4). In spring, Chlorophyta recorded the highest coverage (35.7%) mostly represented by *Ulva lactuca, Caulerpa racemosa*, *Dictyosphaeria cavernosa, Valonia aegagropila* and *Cladophora prolifera*, followed by Rhodophyta (34.3%) with a dominance of *Actinotrichia fragilis* and *Jania rubens.*

**Table 4:**
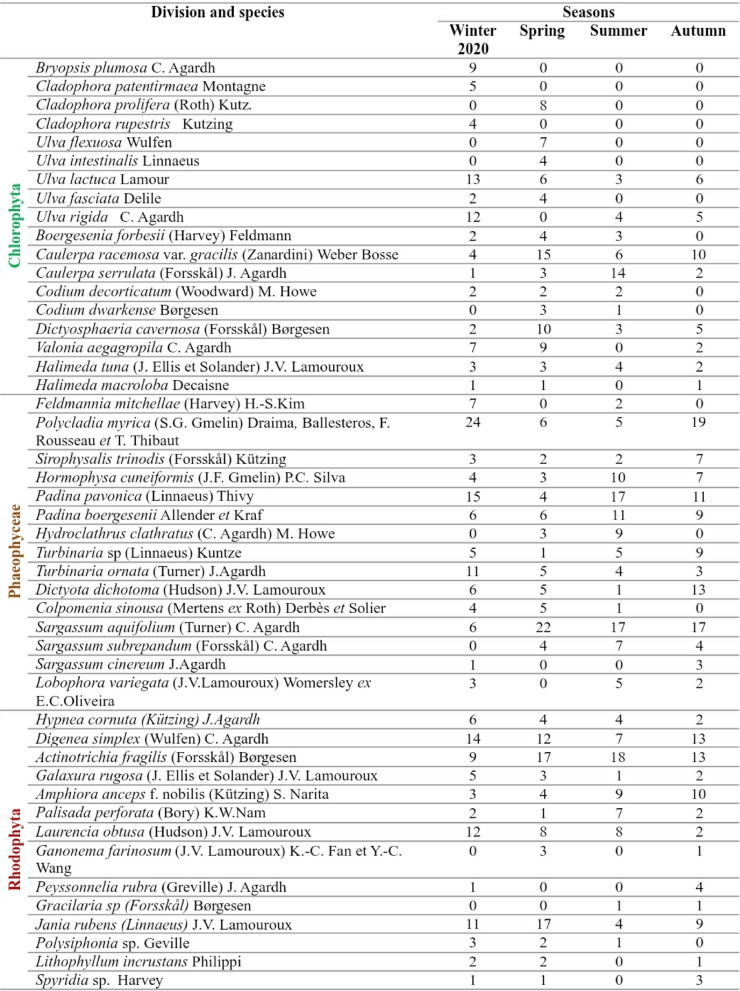
Species list and annual average cover (%) at the different seasons

**Fig. 8:**
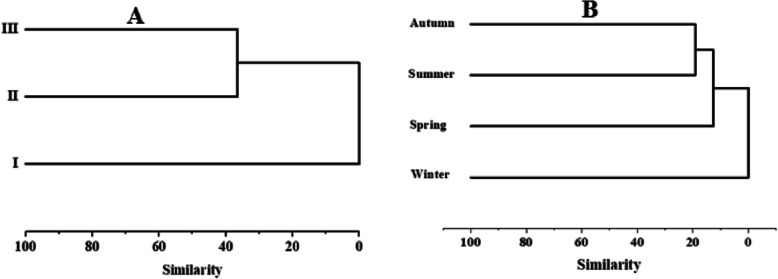
Dendrogram produced by cluster analysis of seaweeds coverage at **A**: sites and **B**: seasons

The cluster analysis of seaweeds coverage among sites and seasons showed that site I was separated from other sites, while site II and III exhibited a higher similarity (> 45%) (Fig. 8). As for seasons, the seaweeds diversity formed 2 groups. The first separates winter from the rest of the seasons, while the second forms two sub-clusters. Autumn and summer were detached into sub-group with 20% similarity.

#### Spatial and temporal variations of dominant seaweeds species

**Fig. 9:**
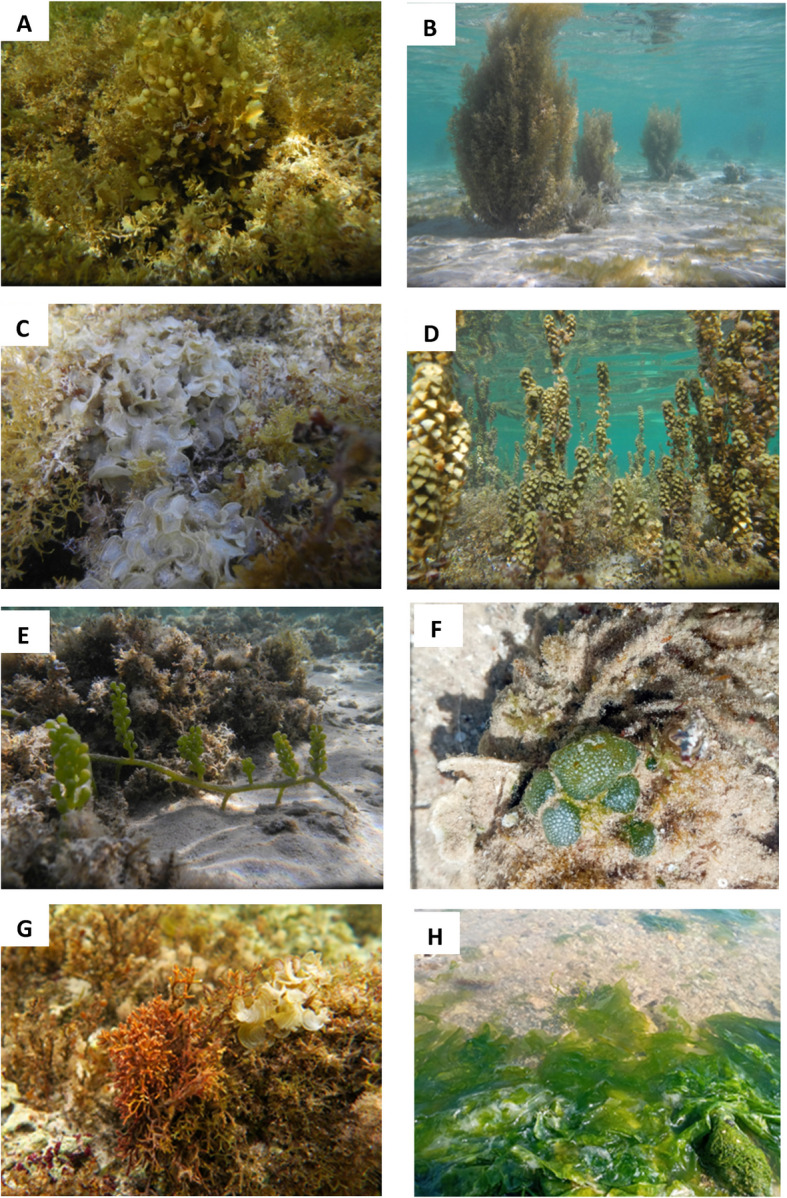
Representative underwater photographs of algal assemblages at the study sites: Sargassum sp. **A**, Polycladia sp. **B**, Padina sp. **C**, Turbanira **D**, Caulerpa sp. **E**, Dictyosphaeria sp. **F**, Actinotrichia sp. **G**, and Ulva sp. **H**

According to the results in (Tables 2 and 4), the most dominant seaweeds species during the study period were *Ulva lactuca*, *Caulerpa racemosa* and *Dictyosphaeria cavernosa* from Chlorophyta, *Padina pavonica*, *Turbinaria ornata*, *Polycladia myrica* and *Sargassum aquifolium* from Phaeophyceae and *Actinotrichia fragilis*, *Digenea simplex* and *Jania rubens* from Rhodophyta (Fig. 9). These species were therefore selected to study their spatial and temporal patterns.

**Fig. 10:**
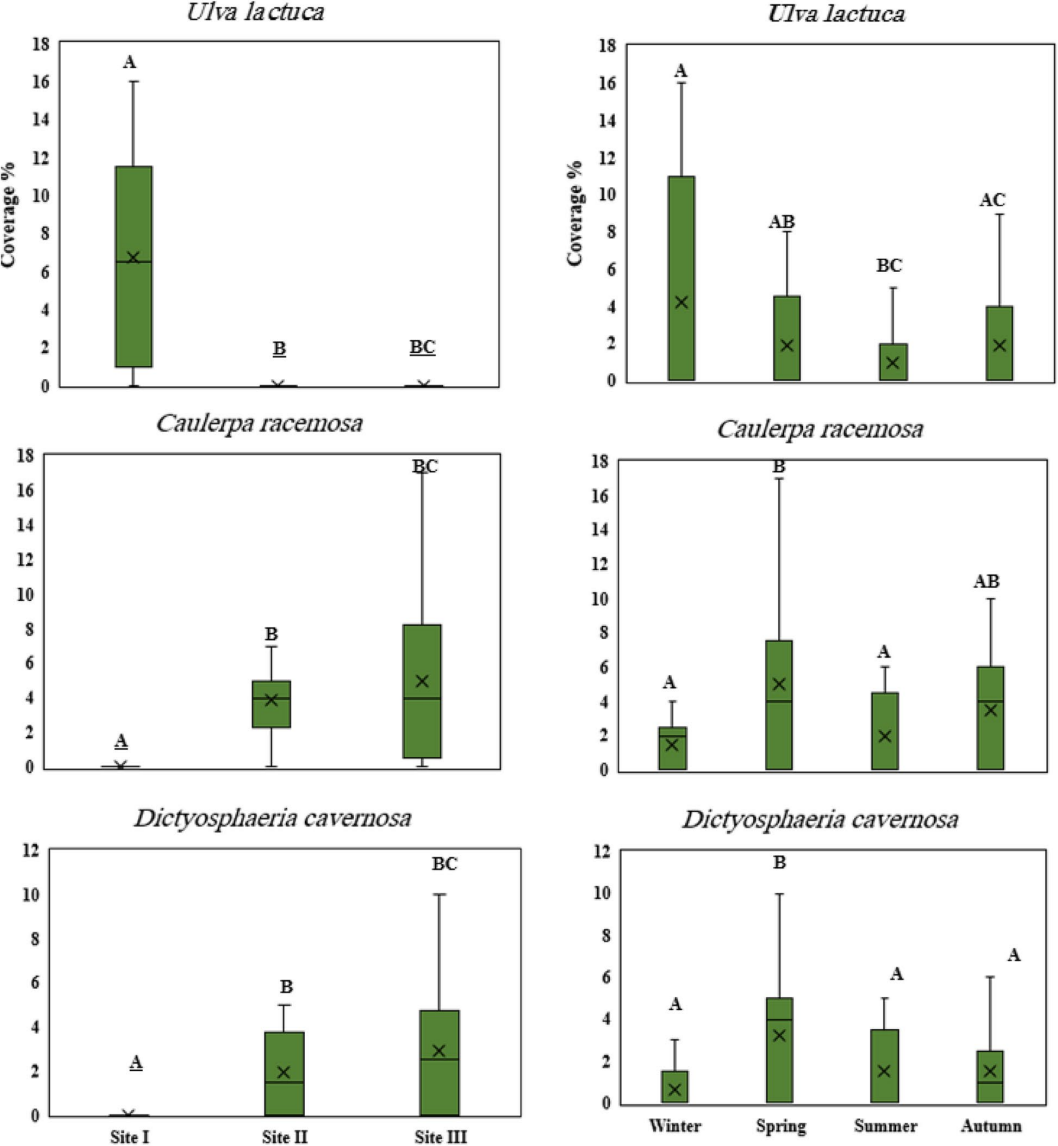
Boxplots of average percentage cover of dominant Chlorophyta species at three study sites during the four seasons of 2022. The capital letters: **A**, **B** and **C** illustrate the similarity or difference between means at *p* ≤ 0.05

During the study, *U. lactuca* was only found at site I with a maximum cover of 16% and inter-quartile range of 10.5% during winter, followed by spring with a maximum cover of 9% (Fig. 10). *C. racemosa* and *D. cavernosa* had the highest median percentage cover (4% and 2.5%, respectively) at site III. Spring had the most variable cover compared to other seasons for both seaweed species.

**Fig. 11:**
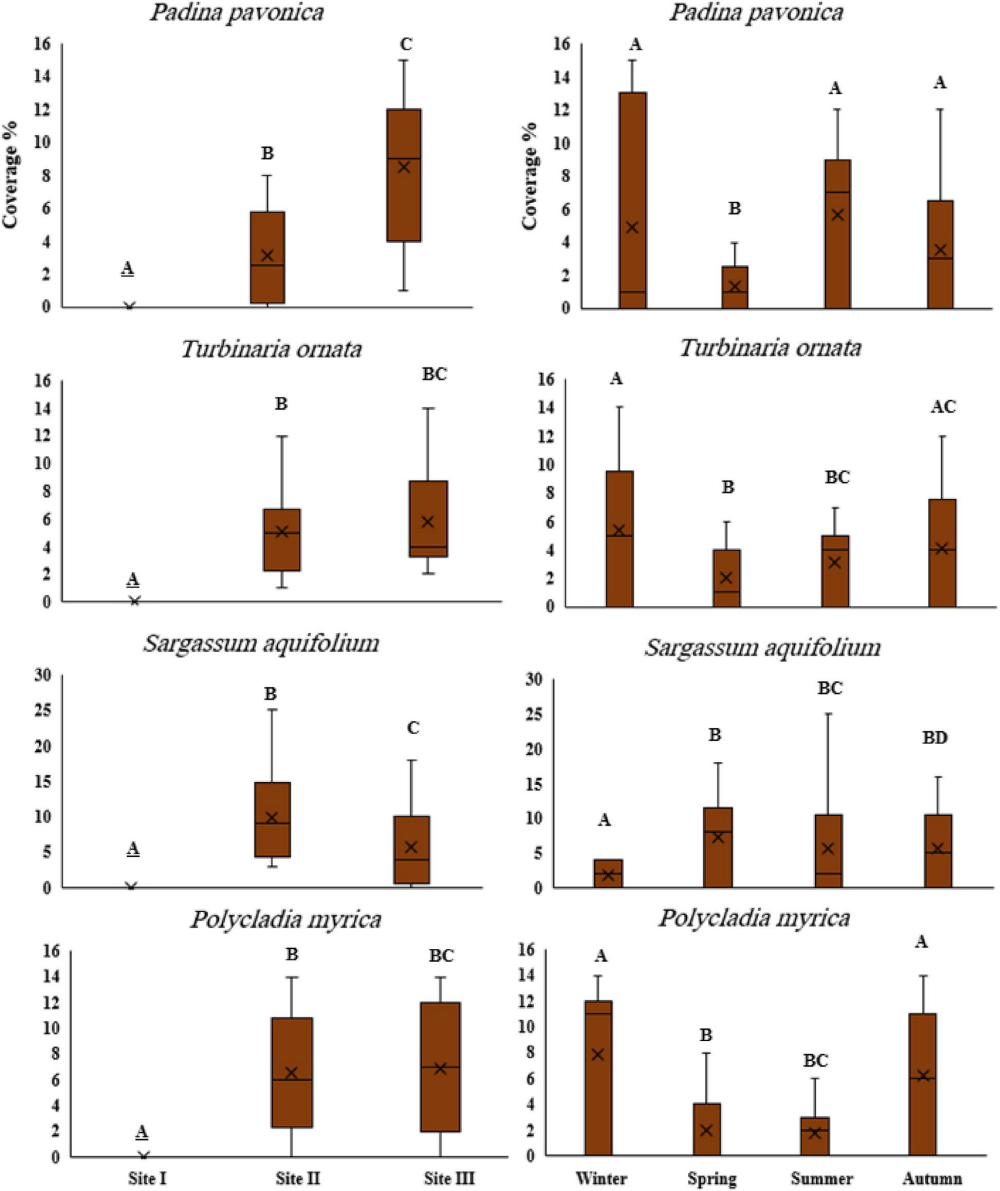
Boxplots of average percentage cover of the dominant Phaeophyceae species at three study sites during the four seasons of 2022. The capital letters: **A**, **B**, **C** and **D** illustrate the similarity or difference between means at *p* ≤ 0.05

Boxplots for brown algae varied among sites and seasons (Fig. 11)*. P. pavonica* coverage showed a maximum median (9%) during summer at site III, followed by site II (2.5%). For the coverage of *T. ornate,* the highest median and variability were during winter (5%) at site III, while the least median was during spring. Site II had the highest median value of *S. aquifolium* (9%) during spring, followed by site III (4%) during summer. The boxplots of *P. myrica* showed that sites II and III had close median values (6% and 7%, respectively) with highest median and variability during winter and autumn (11% and 6%, respectively).

**Fig. 12:**
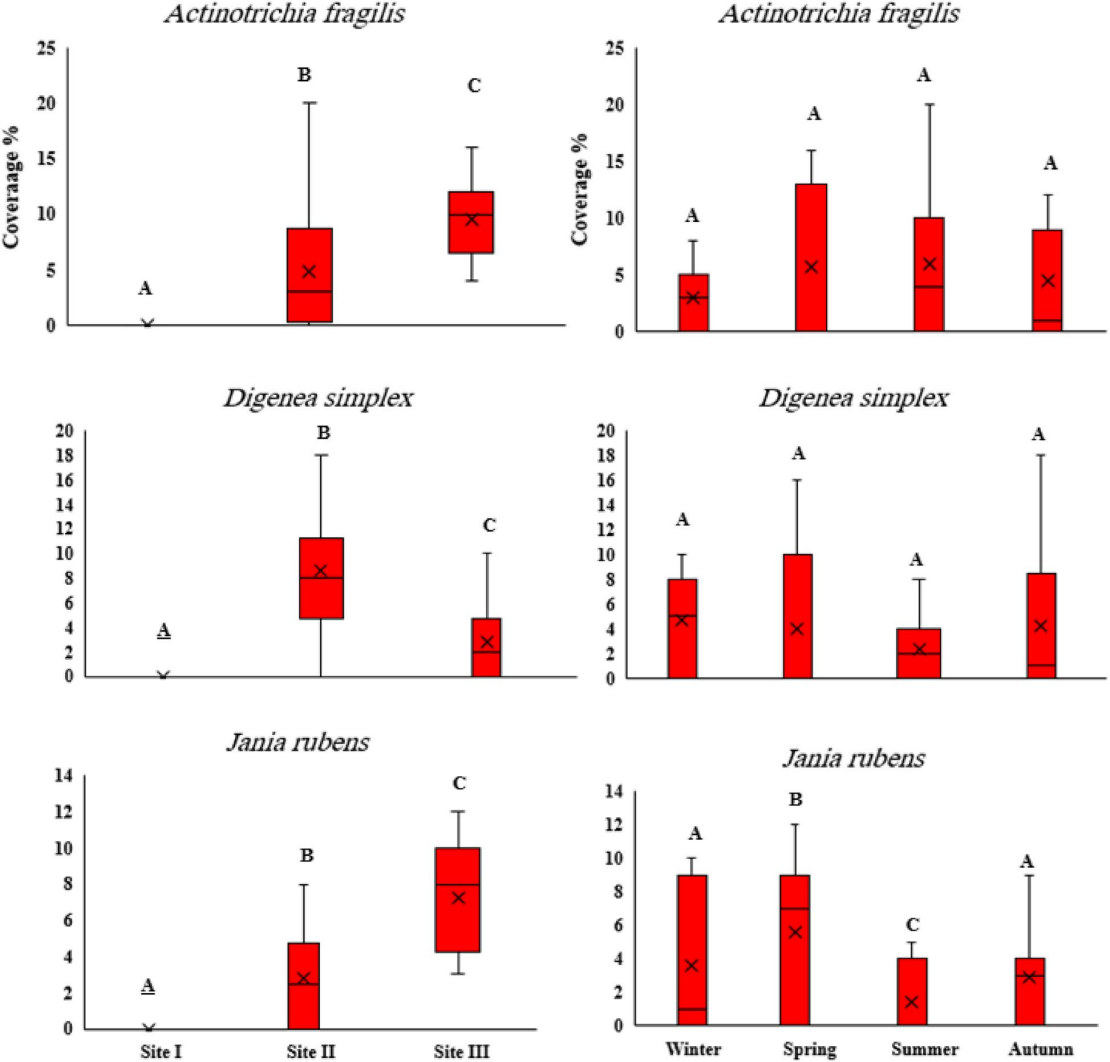
Boxplots of average percentage cover of dominant Rhodophyta species at three study sites during the four seasons of 2022. The capital letters: A, B and C illustrate the similarity or difference between means at *p* ≤ 0.05

Site III recorded the highest median for *A. fragilis* (10%) in summer, while *D. simplex* had the highest median at site II (8%) during winter (Fig. 12). For both seaweeds, the highest inter-quartile range (13% and 10%, respectively) was observed in spring. The length of the boxplot of *J. rubens* showed that the variability of coverage in spring (7%) is higher than in other seasons at site III. In contrast, the least median value (2.5%) and variability were found at site II during summer. ANOVA results indicated a significant difference in the cover distribution between the selected seaweeds among sites and seasons (*p* < 0.05) except for *U. lactuca*, *A. fragilis* and *D. simplex* which showed no significant difference between seasons (*p* > 0.05).

#### Environmental correlation

Correlations between the dominant seaweeds'coverage and each environmental parameter are presented in Table 5. The coverages of *C. racemosa*, *P*. *pavonica* and S. aquifolium showed a positive correlation with water temperature, whereas the coverages of *U. lactuca* and *P. Myrica* displayed a negative correlation. Salinity had a positive effect on the abundance of *C. racemose P. pavonica*, *S. aquifolium* and *P. myrica* while it was negatively correlated with *D. simplex* coverage. Only *P*. *pavonica*, S. aquifolium and *A. fragili* were positively correlated to pH values. The presence of nutrient salts positively influenced the coverage of the majority of the seaweeds except for *T. ornata* and S*. aquifolium*, where they were negatively correlated with nitrate and phosphorus, respectively.

**Table 5:** Correlations between the coverage of selected seaweeds and the environmental parameters; bolded correlation coefficients are significant at *p* ≤ 0.05

Dominant species	Physico-chemical parameters							
	Air T	Water T	pH	Salinity	NH_4_	NO_3_	NO_2_	PO_4_
***U. Lactuca*** ***C. racemosa*** ***D. cavernosa*** ***P. pavonica*** ***T. ornata*** ***S. aquifolium*** ***P. myrica*** ***A. fragilis*** ***D. simplex*** ***J. rubens***	0.403 0.169 0.268 0.171 -0.089 0.252 -0.410 0.328 -0.101 -0.147	-0.850 0.717 0.363 0.638 -0.788 0.690 -0.711 0.525 -0.400 -0.119	0.491 -0.155 0.007 0.628 0.448 0.687 0.406 0.746 -0.425 0. 677	0.146 0.723 -0.096 0.625 -0.015 0.658 0.625 0.579 -0.794 -0.326	0.869 0.729 -0.547 0.316 -0.111 0.572 0.557 -0.424 0.707 0.532	0.804 0.260 -0.572 0.065 -0.733 -0.557 0.538 -0.562 0.682 -0.024	0.436 0.291 0.715 0.255 0.015 0.593 0.645 -0.359 0.579 0.741	0.089 0.642 -.136 0.233 -0.504 -0.578 -0.257 -0.276 0.312 -0.471

## Discussion

In the present study, average surface water temperature fluctuated from 17.9 °C in winter and 32.7 °C in summer, with significant difference between sites. The variation in water temperature in the present study was linked to seasonal changes in air temperature at the different sites with slight increase in southward direction. Close results were obtained by Fahmy et al. [27] who studied the quality of the Egyptian Red Sea coastal waters. The water temperature in the studied sites may be influenced by the intensity of solar radiation, evaporation, season, waves and gain or loss of heat in shallow waters close to the land [36].

The Red Sea surface waters are exceptionally clear and low in nutrients because of the hot and arid climate with lack of river inputs and negligible precipitation [17] and [44]). In our study, Ammonia contents various significantly between sites and seasons with the highest level at site I, which is probably due to the industrial disposals at this site. However, relatively lower concentrations were recorded during autumn and spring, which could be due to the flourishing of phytoplankton that utilize ammonia as a nitrogen source [2].

In the present study, the maximum nitrate concentration was recorded during winter at site II. According to Al-Qutob et al. [9], the winter mixing deepening increased nitrate enrichment into the euphotic zone from deeper water, which indicates the relatively high levels of nitrate at the different sites during winter, On the other hand, the highest concentration of nitrite at the different sites was observed during summer. This can be attributed to increased phytoplankton excretion, ammonia oxidation, nitrate reduction, and nitrogen recycling, as noted by Häse et al*.* [31]. Site I exhibited the highest annual mean concentration of nitrite and PO_4_ due to exposure to various anthropogenic activities, which aligns with the findings of Mofeed and Deyab [39].

Seaweeds vegetation at the three studied sites exhibited high species diversity during the study period. The algal list comprised of 47 species, 18 species belong to Chlorophyta, 15 to Phaeophyceae and 14 to Rhodophyta. Similarly, El-Shoubaky and Kaiser [26] recorded 46 species et al.-Shoaiba coast on the western area of Saudi Arabia, Red Sea. Mohamed et al. [40] recorded 35 species (7 Chlorophyta, 7 Rhodophyta and 21 Phaeophyceae) along the Red Sea from different sites in Hurghada, Safaga, and Al-Quseir. Also, Rashedy et al. [46] studied the variation in macroalgae vegetation in the same sites, recording 55 species, including 20 of Chlorophyta, 16 of Phaeophyceae and 19 of Rhodophyta. On the other hand, some studies at different surrounding habitats reported a relatively higher diversity, like the sector of Shalateen-Halaib (94 sp.) [23] and Zabargad islet (54 sp.) [21]. Compared to previous research findings, the variation in seaweed biodiversity levels could be due to differences in the number of sampling locations, as well as the environmental parameters both in coastal topography, substrate, transparency of waters, anthropogenic impacts and seasonal influences as suggested by Kepel et al. [35].

Ashour et al. [13] recorded the lowest number of species at Suez Bay with the dominance of the same species. El-Manawy and Shafik [24] noted that only *Ulva* and *Enteromorpha* dominated the vegetation in Suez with a significant low species number. They attributed this to the presence of a considerable discharge of industrial pollutants and sewage wastes, which significantly enhance Chlorophyta growth. Our results agreed with those obtained by El-Shoubaky [25], who recorded massive macroalgal mats composed mainly of *Ulva lactuca* and *Ulva fasciata* during spring in the Suez Canal. In the same context, Mofeed and Deyab [39] confirmed the superiority of Chlorophyta vegetation during the entire period of investigation in Suez, with only *Ulva clathrata* and *Ulva lactuca* dominating 76% of the seaweed’s vegetation.

In the present study, seven functional groups were identified. Among these, eight canopy species were found, all from Phaeophyceae with the dominance of *Polycladia myrica* and *Sargassum* sp. Similar results were obtained by Kamal [34] who noted that in canopy algae-dominated areas, waves induce localized loss or stunting of *P. myrica* and *Sargassum*, making gaps in between for turf algae to thrive. Three crustose algae were observed in the present study including, *Lobophora variegate*, *Peyssonelia rubra and Lithophyllum incrustans*. These species are recognized as important framework builders on coral reefs and provide a calcified tissue barrier against erosion, especially on reef crests [56].

Seventeen species of upright fleshy seaweeds were found at sites II and III. Thick mats of *Padina* sp. were observed to colonize any dead coral substrate that emerged from the sediment, in addition, *Hydroclathrus clathratus* formed enlarged elongated globules associated with *Padina* mats in shallow areas during summer. This is in accordance with the findings of Rashedy et al. [46]. As for sheet and filamentous groups such as *Ulva*, *Enteromorpha* and *Cladophora* species, they were only found at site I (Suez). According to Beach et al. [14], seaweeds with filamentous thalli and coarsely branched group are typically more productive and flourish in unstable habitats than thicker and calcareous seaweeds which readily apparent in more constant environments. Finally, the ability of each functional group to prevail in a given location will depend upon the overall characteristics of this location El-Manawy [22].

Species evenness, richness and diversity are informative tools for assessing diversity status where higher values represent healthy environmental quality with stable community composition [50] and [3]). Our observation is in agreement with those of Issa et al. [33], who studied the macroalgal β diversity along the Egyptian coast of the Red Sea in Hurghada, Safaga, and Al Quseir and stated that variation in species richness among sites may be the result of unstable environmental disturbances.

The evenness index varied from 0.84 at site I to 0.92 at Site III, indicating a stable seaweed community across these three sites. This finding aligns with the research conducted by Rashedy et al. [46], who also observed a stable macroalgae community with evenness index values ranging from 0.75 to 0.87 in Hurghada, Safaga, and Al Quseir cities along the Egyptian Red Sea coast. In our study, the Shannon–Weaver index ranged from 2 at site I to 3.3 at site II. According to Kepel et al. [35], a diversity index value below 2 indicates low species diversity, while values of 4 or higher suggest an increasingly diverse community. The community structure at sites II and III indicated moderate diversity and less susceptibility to disturbance compared to site I.

In the present study, the highest coverage of species at Hurghada (site II) and Marsa Alam (site III) was observed during summer and autumn. This is in accordance with the findings of Issa et al. [33] and Rashedy et al. [46] at Hurghada, Safaga, and Al Quseir, Egypt; Ansari [11] at Tabuk Coast, Saudi Arabia,and Ali [7] at Wadi El Gemal, Marsa Alam, Egypt. According to Roy [49], the algal seasonal distribution is probably related to variations in nutrient supply, salinity, temperature, light availability and wave action during different seasons, which may influence the algal abundance in different habitats.

In the present study, *Caulerpa racemosa* and *Dictyosphaeria cavernosa* (Chlorophyta) inhabited all the studied sites except Suez with considerable abundance, reflecting their high ability to adapt to different environmental conditions. Mofeed and Deyab [39] stated that the most important ecological factors that affect the growth rate of *Caulerpa* are light, depth, substrate nutrients, and exposure to waves. According to El-Manawy [22], *C. racemosa* was very common in the Red Sea, mixed with other seaweeds and seagrasses, or found as pure stands inhabiting sands or mud in both lagoons and open coasts. Meanwhile, *Ulva lactuca* was only found with a maximum cover at Suez. This could be attributed to the enriched seawater at this site with phosphorus and nitrogen, as suggested by El-Shoubaky [25].

*Polycladia myrica* and *Turbinaria ornata* were found in all seasons at sites II and III, except for *T. ornata,* which was only found at site III. They had the highest average cover and median in winter. Similarly, Rashedy [45] reported that *P. myrica* was abundant in winter in the south of Hurghada, and its basal parts persisted in the reef flat during winter and autumn. On the other hand, *Padina pavonica* and *Sargassum aquifolium* were commonly found at site III during summer. These species are considered tolerant species (Gardner et al., [29]). This may be attributed to the environmental changes such as high temperatures and salinity that characterize this site. This is in accordance with El-Shoubaky and Kaiser [26], who found the same trend among seasons et al.-Shoaiba coast, Red Sea.

Among the selected red algae, *Digenea simplex* and *Jania rubens* were dominant at both Hurghada and Marsa Alam during winter and spring, respectively, while *A. fragilis* flourished during summer. These results agreed with those obtained by Mohamed et al. [40], who confirmed the abundance of both algae during the same seasons in Hughada and Safaga, Red Sea. This may be due to the relatively low temperature in these seasons, where their abundance decreases with higher temperature [4].

Future studies should include longer-term monitoring (e.g., multiple years) and expand the geographical range to cover additional regions of the Red Sea, as this would provide a more comprehensive understanding of seasonal and spatial variations in macroalgal communities. Furthermore, since some species were identified only to the genus level (e.g., *Turbinaria* sp.) due to challenges in morphological identification, molecular techniques like DNA barcoding should be employed in future studies to achieve higher taxonomic resolution and ensure accurate species identification. Lastly, the study did not explicitly address the potential impacts of human activities, such as tourism, fishing, and coastal development, on macroalgal communities. Future work should evaluate the effects of anthropogenic pressures on seaweed diversity and abundance, providing insights for conservation and sustainable management.

## Conclusion

This study documents a high diversity of species along the study sites. Seasonal analysis revealed that summer and autumn exhibited the greatest species coverage, particularly in Hurghada and Marsa Alam. The physicochemical properties of seawater emerged as a key factor influencing species diversity and community composition. The present study offers a status of the seaweed communities in the studied area, which could be useful for future monitoring and conservation of these vital resources sustainably.

## Data Availability

The datasets used and/or analyzed during the current study available from the corresponding author on reasonable request.
